# ﻿*Lithocarpusdahuensis* (Fagaceae), a new species from Fujian Province based on morphology and genomic data

**DOI:** 10.3897/phytokeys.222.99370

**Published:** 2023-03-17

**Authors:** Miao Zhang, Xiao-Hui Zhang, Shi Shi, Bing-Hua Chen

**Affiliations:** 1 College of Life Sciences, Fujian Normal University, Fuzhou 350117, China; 2 The Public Service Platform for Industrialization Development Technology of Marine Biological Medicine and Products of the State Oceanic Administration, Fujian Key Laboratory of Special Marine Bioresource Sustainable Utilization, Southern Institute of Oceanography, College of Life Sciences, Fujian Normal University, Fuzhou 350117, China; 3 South China Limestone Plants Research Center, College of Forestry and Landscape Architecture, South China Agricultural University, Guangzhou 510642, China

**Keywords:** biodiversity, chloroplast genome, morphology, phylogeny, taxonomy

## Abstract

*Lithocarpusdahuensis*, a new Fagaceae species from Fujian Province, China, is described and illustrated. The new species is morphologically similar to *L.konishii*, but its oblanceolate leaf blade has more pairs of acute teeth on the margin, denser lateral veins, smaller cupules enclosing up to 1/4–1/3 of the nut, and its nut is only half as long as those of *L.konishii*. The plastome of *L.dahuensis* was 161,303 bp in length and displayed the typical quadripartite structure. Phylogenetic analyses distinguished *L.dahuensis* from *L.konishii* with strong support based on whole plastome and nrITS, respectively.

## ﻿Introduction

The subfamily Quercoideae, of the family Fagaceae, consists of seven genera, including *Castanea*, *Castanopsis*, *Chrysolepis*, *Lithocarpus*, *Notholithocarpus*, *Quercus*, and *Trigonobalanus*, containing 1,135 species (The plant list 2022). *Lithocarpus* consists of 341 species, making it the second largest genus after *Quercus*. These species are widely distributed in tropical and sub-tropical broad-leaved evergreen forests throughout East and Southeast Asia, extending to New Guinea (Cannon 2001). The center of diversity is in East to Southeast Asia, with 123 species in China, mainly distributed in Guangdong, Guangxi, and Yunnan (Huang et al. 1999), 58 species in Thailand (Strijk et al. 2014), and 121 species in Vietnam (Ngoc et al. 2022).

Typically, *Lithocarpus* has spirally arranged leaves, which are glabrous, coriaceous, oblong-elliptical to oblong in shape, with the entire margin or with teeth along the margin. Its flowers are white to pale yellow. The male flowers are either solitary or in clusters of three or more, with campanulate or cup-shaped perianths, usually 6-lobed, partially united, and 12 stamens. The female flowers are usually solitary or in clusters of two to five, but only one or two of them are well developed, and they have perianths similar to male flowers, but smaller, and with 12 staminodes (Strijk et al. 2014). The sessile cupules are cup-shaped to discoid, with triangular to rhomboid bracts arranged in a diamond pattern on the cupule surface, enclosing the nuts completely or partially. The nuts are oblate to depressed with a concave or convex scar. Sometimes, the nut scar is concave at the margin but conspicuously convex at the center (Huang et al. 1999).

This paper describes a new species of *Lithocarpus* that was discovered during a field survey in a landscape forest behind the Xuefeng Village in Dahu Town, Minhou County, Fuzhou City in May 2017. It grows in well-preserved native broad-leaved evergreen forests in a valley and has leaves that resemble those of *Quercusengleriana* Seem, which were easily overlooked but were of constant concern. At the end of May 2018, the plants began to bloom and develop erect male inflorescences. By the end of September, fruit-bearing specimens were collected and measured. The newly found species is similar to *L.konishii* from Taiwan but differs in the leaf, cupule, and nut characters, as noted below. Considering the morphological differences, molecular data, and geographical isolation, here we describe it as a new species, *Lithocarpusdahuensis*.

## ﻿Materials and methods

### ﻿Morphological description

The morphological description of the new species was based on the study of specimens collected in 2019 from various locations. A Stereoscopic Zoom Microscope (Carl Zeiss, Axio zoom. v.16, Germany), equipped with an attached digital camera (Axiocam), and a Digital caliper were used to record the sizes of the morphological characters. Field observations provided habitats and phenology for the new species.

### ﻿DNA extraction and sequencing

In this study, total DNA was extracted from fresh leaves of the new species using a DNeasy Plant Mini Kit (Qiagen, Valencia, CA, USA). Purified total DNA of the new species was fragmented, and genome skimming was performed using next-generation sequencing technologies on the Illumina Novaseq 6000 platform. The sequencing was conducted by Berry Genomics Co. Ltd. (Beijing, China) using 150 bp paired-end reads with a 480 bp insert size, resulting in 11.58 GB of reads.

### ﻿Genome assembly, annotation and analysis

The phylogenetic position of the new species was determined through the analysis of nrITS and whole plastome sequences. The nrITS (ITS1-5.8S-ITS2) was assembled using GetOrganelle v1.7.5, with -R of 7 and k-merset of “35, 85, 115”. The embplant_nr library was selected as the reference genome database, then annotated and visualized using Geneious v2021.2.2.

The paired-end reads were filtered and assembled into a complete plastome using a GetOrganelle v1.7.5.0 (Jin et al. 2020) with appropriate parameters, including a K-merset of “21,45,65,85,105”, and a word size of 0.6. Following previous studies, our workflow includes five key steps as well: 1. Mapping reads to seed and assembling seed-mapped reads for parameter estimation; 2. Recruiting more target-associated reads through extending iterations; 3. Conducting de novo assembly; 4. Roughly filtering fortarget-like contigs; 5. Identifying target contigs and exporting all configurations (Bankevich et al. 2012; Camacho et al. 2009; Jin et al. 2020; Langmead and Salzberg 2012). Graphs of the final assembly were visualized by Bandage (Wick et al. 2015) to assess their completeness. Gene annotation was performed using CPGAVAS2 (Shi et al. 2019) and PGA (Qu et al. 2019). The different annotations of protein coding sequences were confirmed using BLASTx. The tRNAs were checked with tRNAscan-SE v2.0.3. Final chloroplast genome map was created using OGDRAW.

### ﻿Phylogenetic analysis

The phylogenetic relationship was constructed using Maximum likelihood (ML) analyses with the combined nrITS sequence. In total, 92 samples of *Lithocarpus*, *Morella*, *Corylus* and *Carpinus* were included in our analysis (Suppl. material 1: table S1). Three species of *Morellarubra*, *Corylusfargesii* and *Carpinuscordata* were used as outgroups (Wu et al. 2022). Each individual locus was aligned using MAFFT 7.310 (Katoh and Standley 2013) with default settings. All missing data were treated as gaps. The best nucleotide substitution model according to the Bayesian Information Criterion (BIC) was TNe+R3, which was selected by ModelFinder (Kalyaanamoorthy et al. 2017) implemented in IQTREE v.1.6.8. Maximum likelihood phylogenies were inferred using IQ-TREE (Nguyen et al. 2015) under the model automatically selected by IQ-TREE (‘Auto’ option in IQ-TREE) for 2000 ultrafast (Minh et al. 2013) bootstraps.

To construct a phylogenetic tree based on plastome sequences, a total of 33 plastome sequences of *Lithocarpus*, *Castanea*, *Castanopsis*, *Cyclobalanopsis*, *Fagus*, *Quercus*, *Trigonobalanus*, *Morella*, *Corylus* and *Carpinus* were included in our analysis (Suppl. material 1: table S2). Three species of *Morellarubra*, *Corylusfargesii* and *Carpinuscordata* were used as outgroups (Wu et al. 2022). Each individual locus was aligned using MAFFT 7.310 (Katoh and Standley 2013) with default settings. The best nucleotide substitution model according to the Bayesian Information Criterion (BIC) was K3Pu+F+R5, which was selected by ModelFinder (Kalyaanamoorthy et al. 2017) implemented in IQTREE v.1.6.8. Maximum likelihood phylogenies were inferred using IQ-TREE (Nguyen et al. 2015) under the model automatically selected by IQ-TREE (‘Auto’ option in IQ-TREE) for 2000 ultrafast (Minh et al. 2013) bootstraps. Bayesian Inference phylogenies were inferred using MrBayes 3.2.6 (Ronquist et al. 2012) under the GTR+F+I+G4 model (2 parallel runs, 2000000 generations), in which the initial 25% of sampled data were discarded as burn-in. Phylograms were visualized in iTOLv.5 (iTOL: Interactive Tree Of Life (embl.de)).

### ﻿Genomic comparison with related species

The online tool IRscope (Amiryousefi et al. 2018) was employed to draw the genetic architecture of the IR/SC junctions. Then the sequences of 12 *Lithocarpus* species were aligned using MAFFT7.310 (Katoh and Standley 2013), the nucleotide diversity (Pi value) of single copy genes and intergenic regions was estimated by DnaSP v.6 (Rozas et al. 2003).

## ﻿Results

### ﻿Characteristics of *Lithocarpusdahuensis* plastome

**Figure 1.**
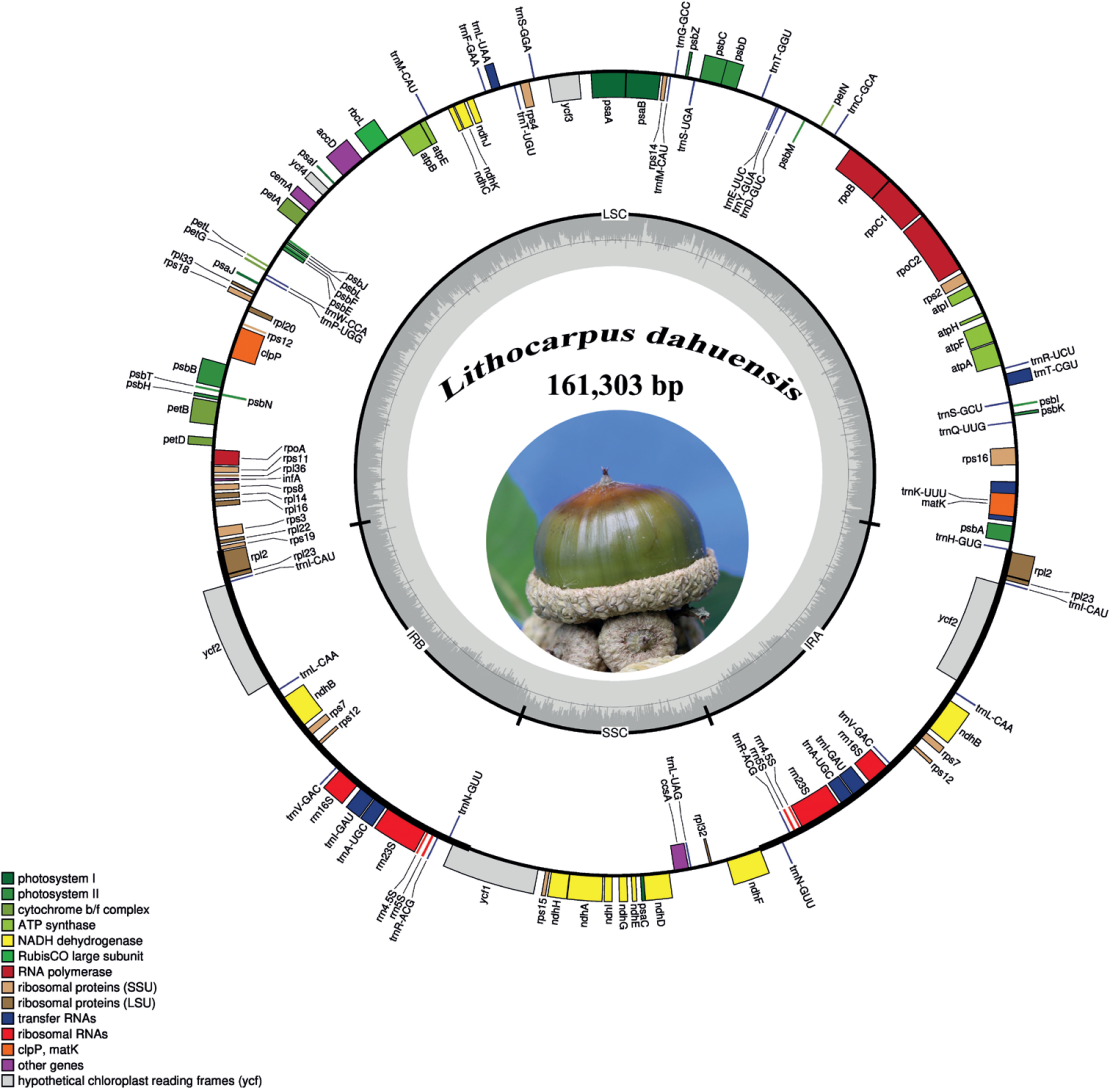
Representative cp genome of *Lithocarpusdahuensis*. Genes drawn inside and outside of the circle are transcribed in clockwise and counter–clockwise directions, respectively. The colored bar indicates chloroplast gene groups. The dark gray bar graphs inner circle shows the GC content, and the light gray bar graphs show the AT content.

The complete chloroplast genome of *Lithocarpusdahuensis* is 161,303 bp in length (Fig. 1), which exhibits a typical quadripartite structure, comprising a pair of IR regions (25,894 bp) divided by an SSC region (18,956 bp) and an LSC region (90,559 bp). The overall GC content of the genome was 36.75%, while the GC content of LSC, SSC, and IR regions were 34.58%, 30.79%, and 42.71%, respectively. The whole chloroplast genome of *L.dahuensis* encodes 129 genes, consisting of 85 protein-coding genes, 36 transfer RNA (tRNA), and 8 ribosomal RNA (rRNA) genes. Among 129 genes, 6 protein-coding genes (*ndhB*, *rpl2*, *rpl23*, *rps*12, *rps*7, *ycf2*), 7 tRNA and 4 RNA genes were duplicated in the genome (Table 1). Altogether, 10 protein-coding genes and 5 tRNA genes contained the intron, in which two genes (*clpP* and *ycf3*) harbored a double intron. The annotated plastome was deposited in GeneBank (accession number OP954095).

**Table 1.** Gene contents in the plastid genome of *Lithocarpusdahuensis*.

Category, group of genes	Gene names
Photosynthesis:	
Subunits of photosystem I	*psaA*, *psaB*, *psaC*, *psaI*, *psaJ*
Subunits of photosystem II	*psbA*, *psbB*, *psbC*, *psbD*, *psbE*, *psbF*, *psbH*, *psbI*, *psbJ*, *psbK*, *psbL*, *psbM*, *psbN*, *psbT*, *psbZ*
Subunits of NADH dehydrogenase	ndhA*, ndhB*(2), *ndhC*, *ndhD*, *ndhE*, *ndhF*, *ndhG*, *ndhH*, *ndhI*, *ndhJ*, *ndhK*
Subunits of cytochrome b/f complex	*petA*, petB*, *petD*, *petG*, *petL*, *petN*
Subunits of ATP synthase	*atpA*, *atpB*, *atpE*, atpF*, *atpH*, *atpI*
Large subunit of rubisco	*rbcL*
Subunits photochlorophyllide reductase	–
Self-replication:	
Proteins of large ribosomal subunit	*rpl14*, *rpl16*, rpl2*(2), *rpl20*, *rpl22*, *rpl23(2)*, *rpl32*, *rpl33*, *rpl36*
Proteins of small ribosomal subunit	*rps11*, rps12*(2), *rps14*, *rps15*, rps16*, *rps18*, *rps19*, *rps2*, *rps3*, *rps4*, *rps7(2)*, *rps8*
Subunits of RNA polymerase	*rpoA*, *rpoB*, rpoC1*, *rpoC2*
Ribosomal RNAs	*rrn16S(2)*, *rrn23S(2)*, *rrn4.5S(2)*, *rrn5S(2)*
Transfer RNAs	trnA-UGC*(2), *trnC-GCA*, *trnD-GUC*, *trnE-UUC*, *trnF-GAA*, *trnG-GCC*, *trnH-GUG*, *trnI-CAU(2)*, trnI-GAU*(2), trnK-UUU*, *trnL-CAA(2)*, trnL-UAA*, *trnL-UAG*, *trnM-CAU*, *trnN-GUU(2)*, *trnP-UGG*, *trnQ-UUG*, *trnR-ACG(2)*, *trnR-UCU*, *trnS-GCU*, *trnS-GGA*, *trnS-UGA*, trnT-CGU*, *trnT-GGU*, *trnT-UGU*, *trnV-GAC(2)*, *trnW-CCA*, *trnY-GUA*, *trnfM-CAU*
Other genes:	
Maturase	*matK*
Protease	clpP**
Envelope membrane protein	*cemA*
Acetyl-CoA carboxylase	*accD*
c-type cytochrome synthesis gene	*ccsA*
Translation initiation factor	*infA*
Genes of unknown function:	
Conserved hypothetical chloroplast ORF	*ycf1*, *ycf2(2)*, ycf3**, *ycf4*

Notes: Gene*: Gene with one introns; Gene**: Gene with two introns; Gene(2): Number of copies of multi-copy genes.

### ﻿Comparative analysis of the plastomes

The plastome of *Lithocarpusdahuensis* was compared to those of the other 11 *Lithocarpus* species. The plastome size of these species is very similar (Table 2), ranging from 161,020 bp for *L.balansae* to 161,974 bp for *L.polystachyus*. These genomes displayed a typical circular quadripartite structure consisting of a pair of IR regions (25,606 bp to 25,899 bp) separated by an LSC region (90,407 bp to 90,731 bp) and an SSC region (18,239 bp to 19,255 bp) (Table 2). The overall GC content was identical (~36.7%) across all compared plastomes, and was clearly higher in the IR region (~42.7%) than in the other regions (LSC ~34.5% and SSC ~30.8%), possibly because of the high GC content of the rRNA that was located in the IR regions.

**Table 2.** Statistics on the basic features of the plastid genomes of *Lithocarpusdahuensis* and related taxa.

Species	Accession No.	Number of Genes			Length (bp)				GC Content (%)			
		PCGs	tRNA	rRNA	Total	LSC	SSC	IR	Total	LSC	SSC	IR
*Lithocarpusdahuensis*	OP954095	79	29	4	161,303	90,559	18,956	25,894	36.75	34.58	30.79	42.71
*Lithocarpuskonishii*	ON422319.1	80	30	4	161,385	90,660	18,927	25,899	36.77	34.61	30.83	42.71
*Lithocarpuslitseifolius*	NC_063927.1	79	29	4	161,322	90,551	18,977	25,897	36.73	34.57	30.71	42.71
*Lithocarpushancei*	MW375417.1	80	31	4	161,304	90,585	18,959	25,897	36.72	34.57	30.68	42.70
*Lithocarpuslonginux*	NC_062048.1	80	29	4	161,420	90,407	19,255	25,879	36.76	34.58	31.01	42.71
*Lithocarpusdealbatus*	NC_063459.1	80	29	4	161,476	90,731	18,987	25,879	36.75	34.58	30.88	42.71
*Lithocarpusbalansae*	KP299291.1	80	31	4	161,020	90,596	19,160	25,632	36.71	34.53	30.83	42.77
*Lithocarpusfenestratus*	OM112300.1	80	31	4	161,184	90,524	19,052	25,804	36.73	34.55	30.78	42.74
*Lithocarpuspolystachyus*	OL569560.1	75	27	4	161,974	90,523	18,239	26,606	36.70	34.58	30.86	42.30
*Lithocarpuscleistocarpus*	OM112296.1	80	31	4	161,178	90,558	19,096	25,762	36.78	34.61	30.83	42.78
*Lithocarpusobscurus*	OM112297.1	80	31	4	161,349	90,616	18,969	25,882	36.79	34.63	30.92	42.71
*Lithocarpusglaber*	MZ750954.1	81	30	4	161,171	90,466	18,939	25,883	36.75	34.61	30.71	42.71

A chloroplast genome identification analysis was performed on the 12 *Lithocarpus* species described above, with the *Lithocarpusdahuensis* chloroplast genome used as a reference (Suppl. material 1: fig. S1). We identified a considerable number of variations in the noncoding cp sequences, including *rbcL-accD* (highest, 0.03019), *rpl20*-*rps12*, *trnK*-*rps16*, *trnF*-*ndhJ* and *ccsA*-*ndhD*, as well as a number of variations in the coding regions, including *accD* (highest, 0.03019), *ycf1*, *rps16*, *ndhF* and *rpl32*. These 10 genes or spacer regions were all in the non-IR region, with *rbcL-accD*, *rpl20-rps12*, *trnK-rps16*, *trnF-ndhJ*, *accD*, *rps16* in the LSC region, and *ccsA-ndhD*, *ycf1*, *ndhF*, *rpl32* in the SSC region (Suppl. material 1: fig. S2). This result is consistent with the highest GC content in the IR region (IR42.71>LSC34.58>SSC30.79 (Table 2).

### ﻿Phylogenetic analysis

**Figure 2.**
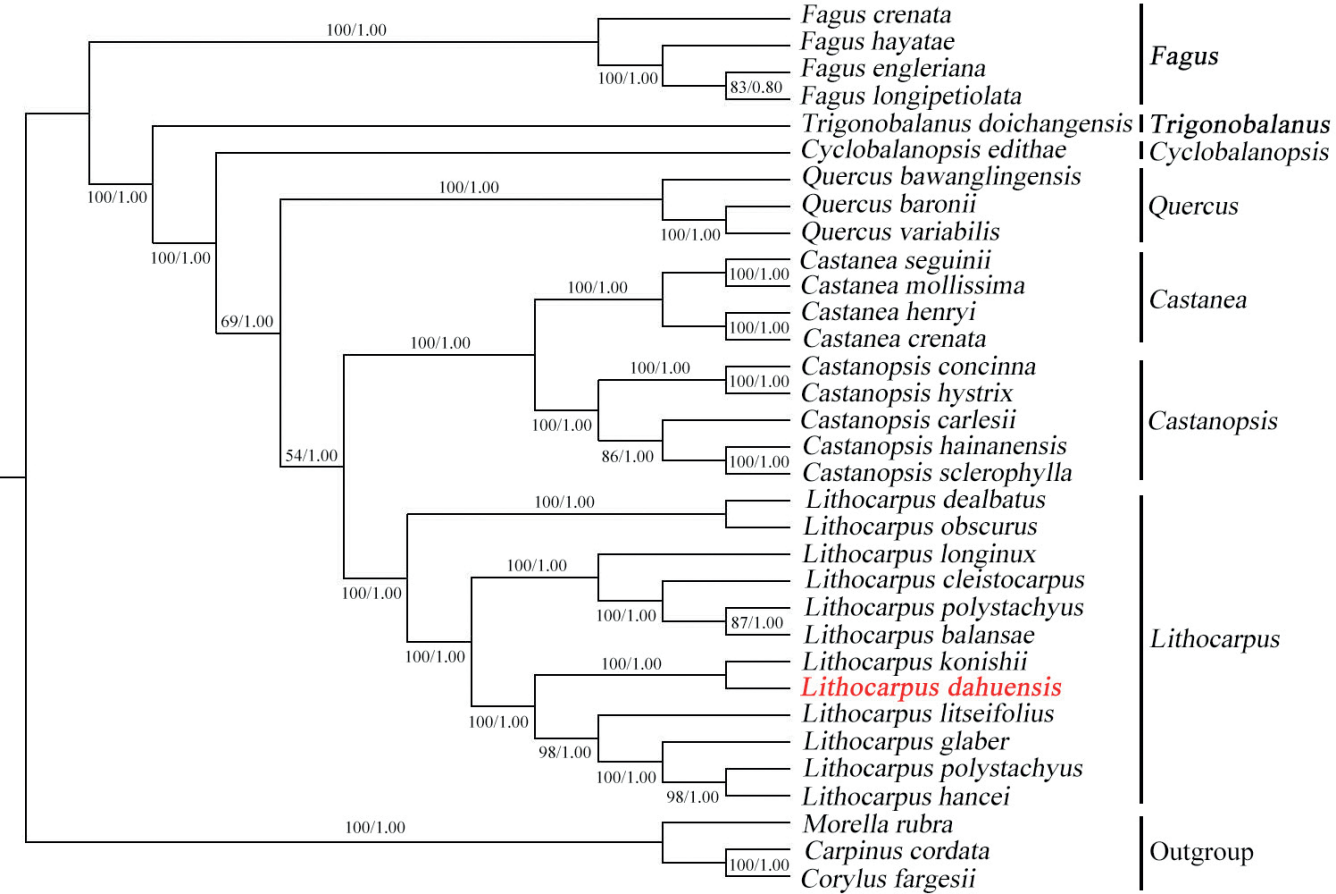
Phylogenetic tree of 33 complete plastid sequences derived from the Fagales species in genus *Lithocarpus*, *Castanea*, *Castanopsis*, *Cyclobalanopsis*, *Fagus*, *Quercus*, *Trigonobalanus*, *Morella*, *Corylus* and *Carpinus*. Numbers above and below branches indicate RAxML (left) bootstrap probabilities (BP) and Bayesian (right) posterior probabilities (PP), respectively. Three species of *Morellarubra* (Myricaceae), *Corylusfargesii* (Betulaceae) and *Carpinuscordata* (Betulaceae) were included as outgroups.

**Figure 3.**
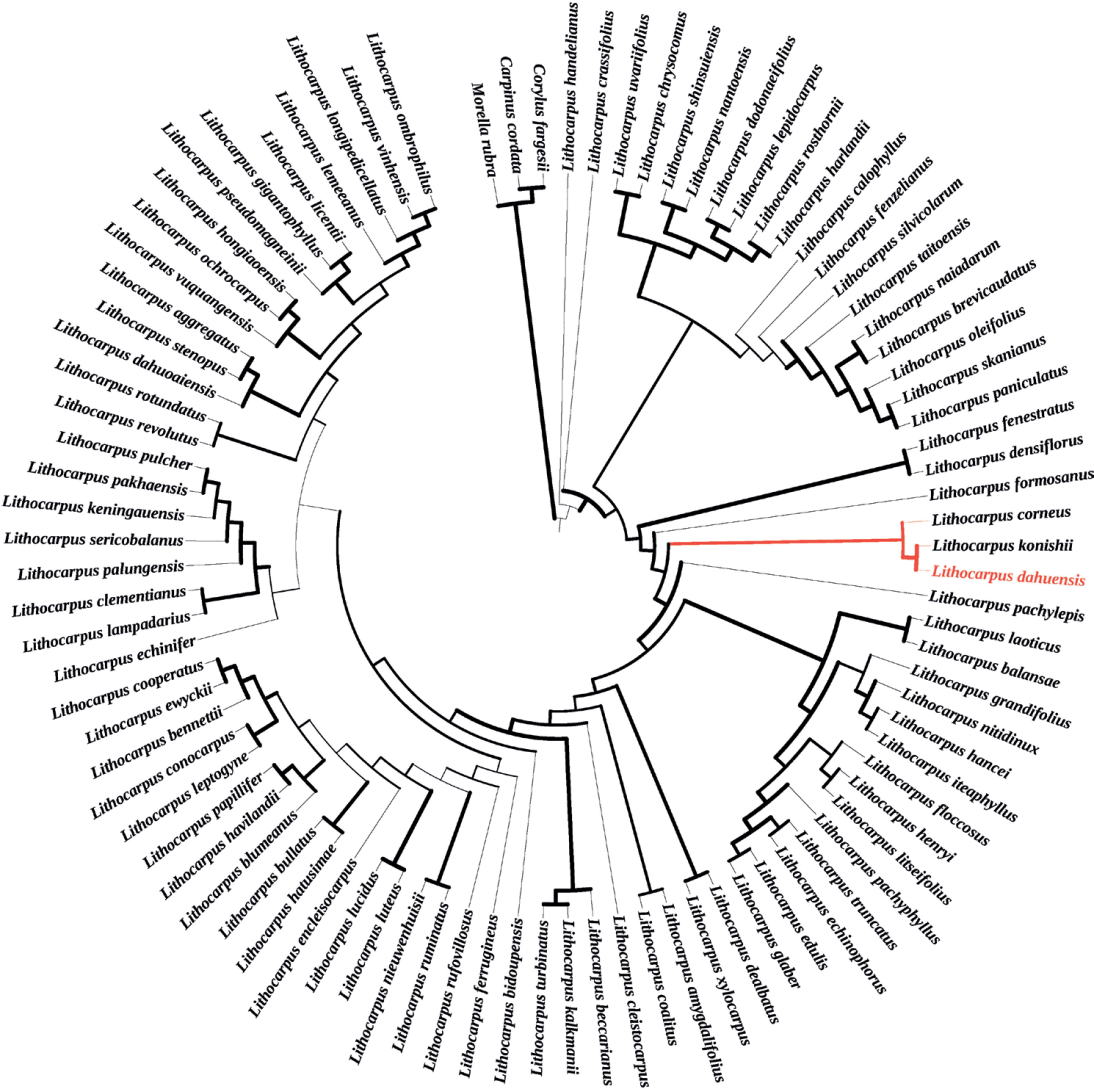
Phylogenetic tree of 92 sequences based on nrITS genes. The boldness of the branches indicates RAxML bootstrap probabilities (BP). Three species of *Morellarubra* (Myricaceae), *Corylusfargesii* (Betulaceae) and *Carpinuscordata* (Betulaceae) were included as outgroups.

The present study confirmed *Lithocarpusdahuensis* as a new species based on phylogenetic analysis based on plastome data, as well as the nrITS sequence. The plastome tree clearly indicated the distinctiveness of *L.dahuensis* from *L.konishii*, with strong support. *Lithocarpusdahuensis* is sister to *L.konishii*, and nested in a clade formed by 4 other *Lithocarpus* species, including *L.litseifolius*, *L.glaber*, *L.polystachyus* and *L.hancei* (Fig. 2). In addition, the phylogenetic analysis based on the nrITS sequence also separates the new species from *L.konishii* with strong support (Fig. 3).

## ﻿Taxonomic treatment

### ﻿ Lithocarpus dahuensis

Taxon classificationPlantaeFagalesFagaceae

H.X.Su, Miao Zhang & B.Hua Chen sp. nov.

F5B25DB8-D4D4-5B55-A645-368D718CCD33

urn:lsid:ipni.org:names:77315865-1

**Figure 4.**
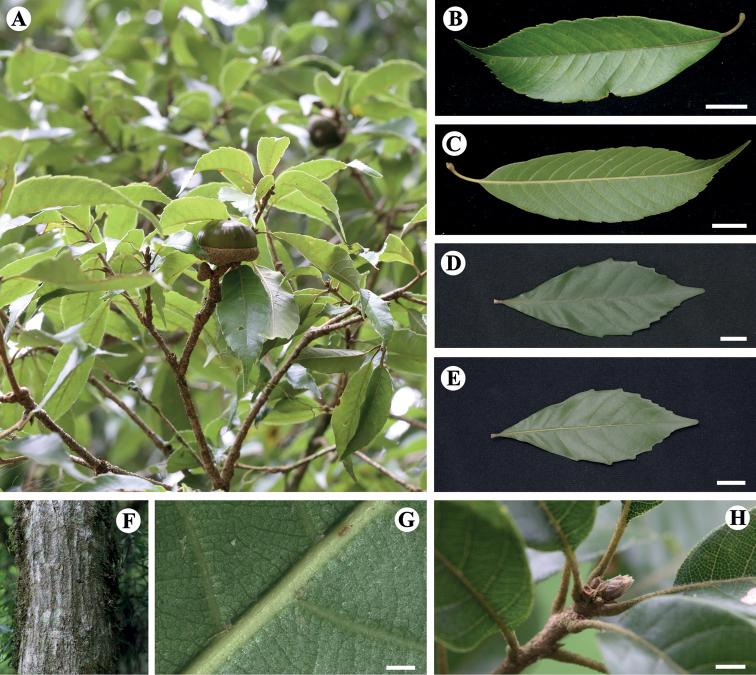
*Lithocarpusdahuensis* H.X.Su, Miao Zhang & B.Hua Chen, sp. nov. **A** fruiting stem with mature cupule **B, C** adaxial and abaxial side of mature leaf,respectively **D, E** adaxial and abaxial side of mature leaf of *Lithocarpuskonishii* (Hayata) Hayata, respectively (photographed by Shi Shi) **F** bark **G** lower young leaf surface, showing abaxially hairy **H** petiole and buds, showing grayish yellow short hairs. Scale bars: 1 cm (**B–E**); 1 mm (**G**); 2 mm (**H**).

**Figure 5.**
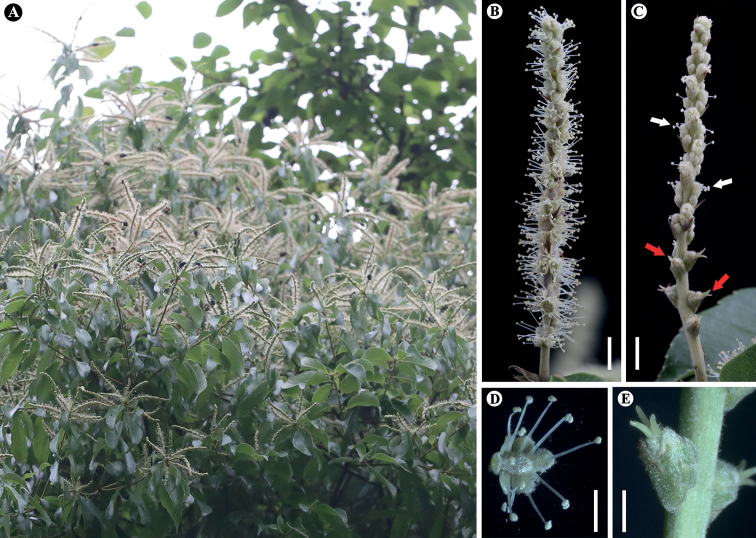
*Lithocarpusdahuensis* H.X.Su, Miao Zhang & B.Hua Chen, sp. nov. **A** branch with mature male and androgynous inflorescences **B** male inflorescences **C** androgynous, showing female flowers borne on basal part of inflorescences (red arrows) and male flowers (white arrows) **D** male flower **F** female flower. Scale bars: 5 mm (**B, C**); 2 mm (**D, E**).

**Figure 6.**
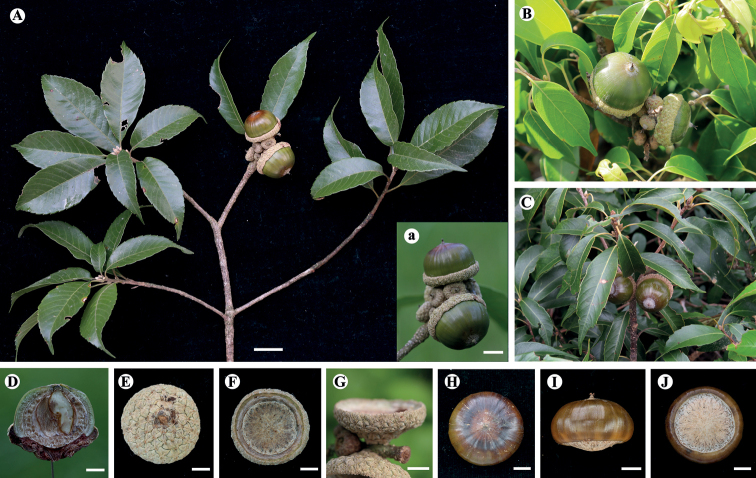
*Lithocarpusdahuensis* H.X.Su, Miao Zhang & B.Hua Chen, sp. nov. **A** branch with mature cupule **a** infructescence, showing mature cupule enclosing 1/4 of nut **B, C***Lithocarpuskonishii* (Hayata) Hayata (photographed by Shi Shi and Jin-Long Zhang from Hainan and HongKong, respectively) **D** vertical section of mature fruit **E–G** outside, inside and side view of the cupule, respectively **H–J** top, side and bottom view of mature nut, respectively. Scale bars: 2 cm (**A**); 1 cm (**a**); 5 mm(**D–J**).

**Figure 7.**
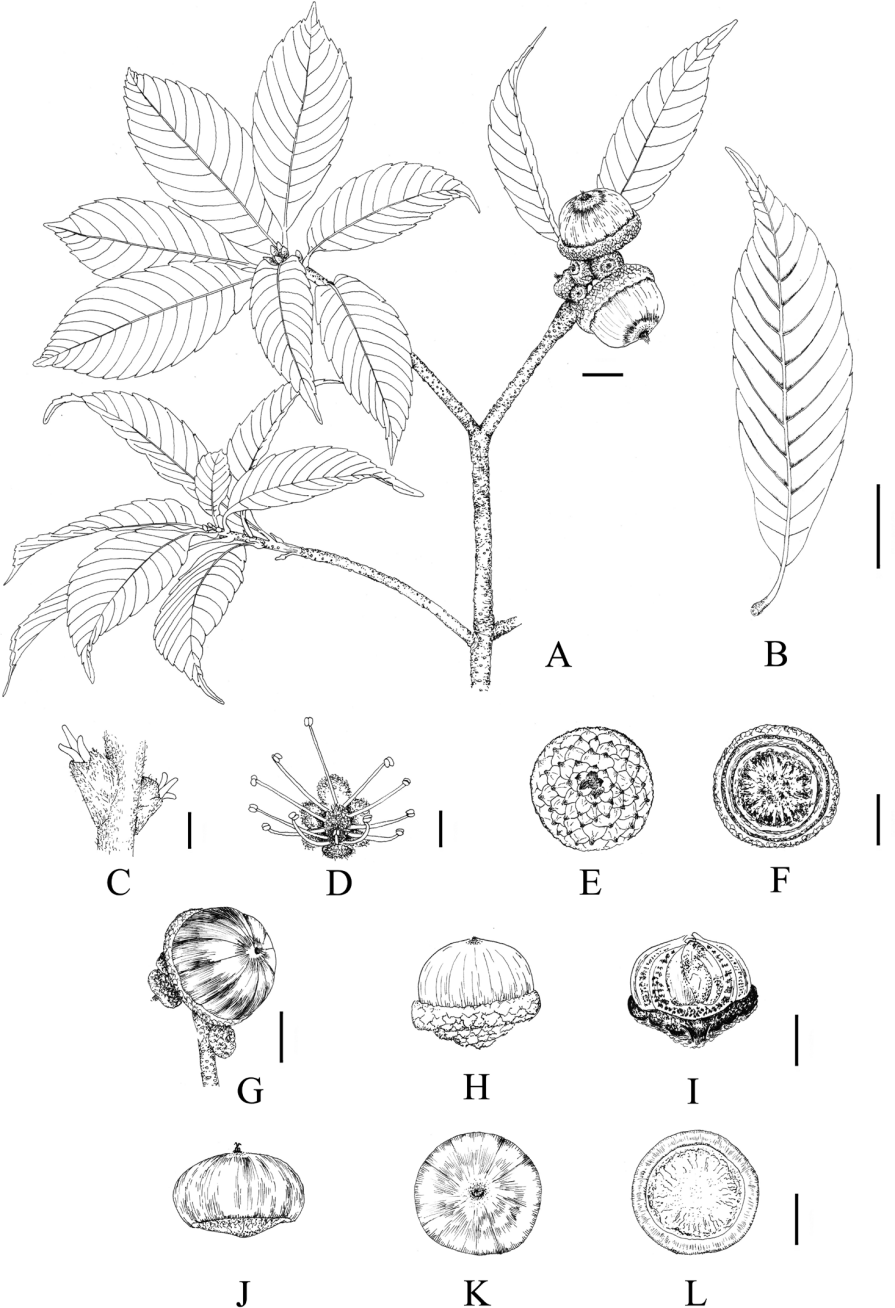
*Lithocarpusdahuensis* H.X.Su, Miao Zhang & B.Hua Chen, sp. nov. **A** fruiting branch with mature cupule **B** abaxial side of mature leaf **C** female flower **D** male flower **E, F** outside and inside view of the cupule, respectively **G** infructescence, mature cupule usually solitary **H** mature cupule enclosing 1/4 of nut **I** vertical section of mature fruit **J–L** side, top and bottom view of mature nut, respectively. Scale bars: 1 cm (**A, B, E–I**); 1 mm (**C, D**).

Figs 4A−C, F−H , 5 , 6A, D−J , 7

#### Diagnosis.

*Lithocarpusdahuensis* differs from *L.konishii* by having an oblanceolate leaf blade with 7–10 pairs of acute teeth on the leaf margin from the second to third lateral veins above the leaf base (compared to 3–6 pairs of obtuse teeth for *L.konishii*), and its lateral veins are numerous and dense, reaching up to 15 pairs; it has 4–10 female flowers, borne singly in the lower part of staminate catkins; the cupules are smaller, encrusting up to 1/4–1/3 of the nut, and the nut is only half as high as those of *L.konishii* (1.4–1.8 *vs.* 1.8–2.4 cm) (Table 3).

**Table 3.** Morphological differences between *Lithocarpusdahuensis* and *L.konishii*.

Characters	*Lithocarpusdahuensis*	*Lithocarpuskonishii*
Leaf margin	Acute teeth; 7–10 pairs	Obtuse teeth; 3–6 pairs
Leaf surface	Glabrous on both upper and lower surfaces, only biaxially retaining fascicled hairs on axil of veins	Glabrous on both upper and lower surfaces, only biaxially retaining fascicled hairs on axil of veins
Leaf shape	Oblanceolate, ovate-elliptic	Ovate, obovate, elliptic, or obovate-elliptic
Leaf blade size (cm)	3.7–9.8 ×1.1–3.2	4.0–9.0 ×1.0–4.0
Petiole length	0.5–1.3 cm long	0.5–1.5 cm long
Number of secondary veins	9–13 (–15) pairs	7–10 (–11) pairs
Fruiting stalk length	Almost sessile	Almost sessile
Cupule	Usually solitary, few in clusters of 2 or 3 (–4), 0.5–0.7 cm high by 2.2–2.7 cm in diam.	Solitary (or 2), 0.7–1.1 cm high by 2.3–3.2 cm in diam.
Cupule outside	Very faintly visible hairs	Faintly visible hairs
Scale arrangement	Imbricate	Imbricate
Nut size	1.4–1.8 cm high by 1.5–2.6 cm in diam.	1.8–2.4 cm high by 2.3–3.3 cm in diam.
Nut enclosure by cupule	Enclosing ca.1/4–1/3 of the nut	Enclosing basal part of nut
Basal scar of the nut	Margin concave but center convex, ca 1.7 cm in diam.	Margin concave but center ± convex, ca 2.0 cm in diam.
Infructescence length	1.5–4.0 cm long	2.0–3.0 cm long

#### Type.

China. Fujian Province, Fuzhou City, Minhou County, Dahu town, Niumu Mountain, forest margins, 26°25'N, 119°3'E, elevation 1035 m, 10 Sep. 2017, B. Hua Chen *CBH02292* (Holotype, FNU, barcode FNU0039021; Isotypes, FNU, barcode FNU0038769).

#### Description.

Trees usually less than 8 m tall, evergreen. Branchlets densely grayish yellow tomentose, soon glabrescent. Bud scales compact, densely covered with grayish yellow silky short hairs. Leaf blade oblanceolate or ovate-elliptic, 3.7–9.8 × 1.1–3.2 cm, leathery, concolorous, apex acuminate to caudate, ca. 9 mm long, base cuneate and inaequilateral, margin with 7–10 acute teeth except basally entire, abaxially with tufts of hairs along veins; midvein adaxially puberulent; secondary veins 7–16 on each side of midvein, adaxially slightly impressed; tertiary veins slender, evident. Petiole 0.5–1.3 cm, tomentose, soon glabrescent. Rachis of inflorescences densely tawny tomentose. Inflorescences male, or androgynous, 2–6, in leaf axils toward base of branchlets or in a dense paniculate cluster on subterminal shoots, erect; rachis of male inflorescences, 5.6 cm long; flowers usually 3 in dichasial clusters; perianth 6-lobed; stamens 12. Female flowers 4–11, borne on basal part of androgynous inflorescences, perianth 6-lobed, styles 3, 3 mm. Infructescences 1.4–4.3 cm; rachis 4.5 mm thick, glabrescent, lenticellate. Cupule usually 1(or in clusters of 2–3–(4)), saucer-shaped, 4.6–7.4 mm × 2.2–2.7 cm, enclosing ca. 1/4–1/3 of nut, wall 1.0–2.5 mm thick; bracts imbricate, broadly triangular, covered with grayish brown, shortly tomentose hairs, midvein ridged. Nut depressed globose, 1.0–1.9 × 1.5–2.6 cm, glabrous, apex flat, wall 3.3–6.9 mm thick and horny; scar 1.5–1.9 cm in diam., margin concave but center convex.

#### Distribution and habitat.

**Figure 8.**
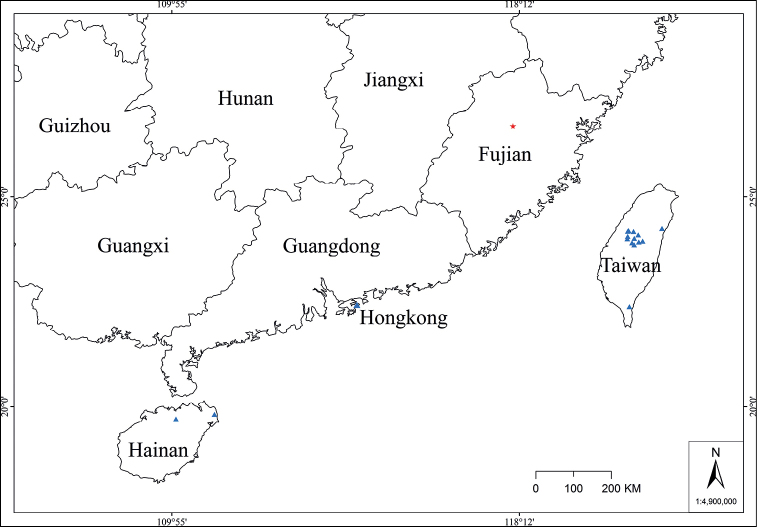
Distribution of *Lithocarpusdahuensis* and *L.konishii* in China. Legend: (red star) *L.dahuensis*, (blue triangle) *L.konishii*.

*Lithocarpusdahuensis* is only found in Dahu town, Minhou County, Fujian, China (Fig. 8), where it grows in valleys of subtropical evergreen broad-leaved forest. Many other plants grow in the surrounding habitat, whose tree layer includes *Castanopsiseyrei* (Champ. & Benth.) Tutcher (Fagaceae), *Quercussessilifolia* Blume (Fagaceae), *Schimasuperba* Gardner & Champ. (Theaceae), *Semiliquidambarchingii* (F.P.Metcalf) H.T.Chang (Altingiaceae), *Ilexelmerrilliana* S.Y.Hu (Aquifoliaceae), *Dendropanaxdentiger* (Harms) Merr. (Araliaceae) and others; the shrub layer includes *Rhododendronovatum* (Lindl.) Planch. (Ericaceae), *Syzygiumbuxifolium* Hook. & Arn. (Myrtaceae), *Linderaaggregata* (Sims) Kosterm. (Lauraceae), *Symplocosstellaris* Brand (Symplocaceae), *Ardisiacrispa* (Thunb.) A. DC. (Primulaceae), *Ilexasprella* (Hook. & Arn.) Champ. ex Benth. (Aquifoliaceae), *Cleyerajaponica* Thunb. (Pentaphylacaceae), *Pyrulariaedulis* (Wall.) A. DC. (Santalaceae), *Oligostachyumoedogonatum* (Z.P. Wang & G.H.Ye) Q.F.Zhang et K.F.Huan (Poaceae), *Ilexserrata* Thunb. (Aquifoliaceae), *Rubusbuergeri* Miq.(Rosaceae), *Rubusimpressinervus* F.P.Metcalf (Rosaceae), *Ligustrumsinense* Lour. (Oleaceae), *Clerodendrumcyrtophyllum* Turcz. (Lamiaceae), *Erythroxylumsinense* Y.C.Wu (*Erythroxylaceae*) and others; the vegetation layer includes *Dicranopterispedata* (Hout.) Nakaike (Gleicheniaceae), *Hypolepispunctata* (Thunb.) Mett. (Dennstaedtiaceae), *Diplopterygiumchinense* (Rosenst.) De Vol (Gleicheniaceae), *Woodwardiajaponica* (L. f.) Sm. (Blechnaceae), *Lobeliasessilifolia* Lamb. (Campanulaceae), *Oenanthelinearis* Wall. & DC. (Apiaceae), *Melastomadodecandrum* Lour. (Melastomataceae), *Carexperakensis* C.B.Clarke (Cyperaceae), *Anoectochilusroxburghii* (Wall.) Lindl. (Orchidaceae), Tainia dunnii Rolfe (Orchidaceae) and others; the interlayer plants include *Trachelospermumbrevistylum* Hand.-Mazz. (Apocynaceae) and others.

#### Phynology.

Florescence May to June, fruiting season September to October of the following year.

#### Etymology.

Chinese name: 大湖柯 (da hu ke). The epithet dahuensis (大湖) refers to Dahu town, Minhou County, Fujian Province where this new species was found.

#### Taxonomic notes.

The following morphological characteristics were used to classify the species, including the acute teethed leaf blade margins, a concave nut scar, and cupules that do not completely enclose the nut. There are four other plants share similar characteristics with *L.dahuensis*, including cupule encrustation (the cupule base is sessile, encasing the base of the nut or about half of it) and a fruit umbilicus (the surrounding margin of the fruit umbilicus is clearly concave), the differences between which are shown in the key.

#### Conservation status.

During our fieldwork from 2017 to 2022, fruit-bearing large trees of *Lithocarpusdahuensis* were only found in the landscape forest of the Xuefeng village valley, Dahu town, Minhou County, Fujian Province, China. They were also found in the surrounding secondary coniferous and broad-leaved mixed forest, but these were mostly small trees that sprouted after the large trees were felled and did not bear fruit. As the location was discovered to be the only known position, we suggest its placement in the Data Deficient category of IUCN (2022).

## ﻿Discussion

Phylogenetic analysis was completed on the whole chloroplast genomes, and nrITS sequences of the Fagaceae species. Based on the well-supported phylogenetic trees (Figs 2, 3), *Lithocarpusdahuensis* is a new species most closely related to *L.konishii*. It is worth noting that *L.konishii* is found mainly in the central and southern regions of Taiwan, the eastern regions of Hainan and Hong Kong, all of which are islands. Nevertheless, *L.dahuensis* was found to be endemic in the mountains of central Fujian, separated by a strait and over 385 km from *L.konishii*. *Lithocarpuskonishii* is found at altitudes between 100–1150 m, usually 500–700 m (Huang et al. 1999), whereas *L.dahuensis* only occurs at altitudes above 1000 m.

The new species has an overall morphology similar to *Lithocarpuskonishii* from Taiwan (Huang et al. 1999). However, there are some obvious differences, especially in the morphology of the leaf, cupules and nuts, such as the oblanceolate leaf blade of *L.dahuensis* has up to 15 pairs of dense lateral veins and 7–10 pairs of acute teeth on the leaf margin from the 2^nd^–3^rd^ lateral veins above the leaf base, whereas *L.konishii* has 5–8 pairs of obtuse teeth from the 3^rd^ lateral vein above the leaf base. *L.dahuensis* has a smaller cupule that encloses up to 1/4–1/3 of the nut, and its nut is only half as long as those of *L.konishii* (1.4–1.8 *vs.* 1.8–2.4 cm).

As a result of its simple, stable genetic structure and ease of sequencing, the chloroplast genome has become increasing popular for species identification, phylogeny reconstruction, demographic history tracing and species divergence studies (Liu et al. 2020). However, genomic information on *Lithocarpus*, particularly the complete chloroplast genome in the NCBI database was very limited. In the current study, we sequenced and assembled the whole chloroplast genome of the new *Lithocarpus* species and found that the chloroplast genome of *L.dahuensis* was 161,303 bp in length, within the expected range (107–218 kb) of most angiosperm chloroplast genomes (Heslop-Harrison 2017), and it had the typical quadripartite structure (Jansen et al. 2005; Daniell et al. 2016). The GC content of *L.dahuensis* chloroplast genome was low (36.75%), which is similar to that reported from other Fagaceae genomes (Yang et al. 2017; Hinsinger and Strijk 2019; Pang et al. 2019).

The chloroplast genome of the new species was compared with the other members of *Lithocarpus* to understand its structural variations and rearrangements. The results showed that all 12 *Lithocarpus* plastomes were remarkably similar in terms of size, genes, and genome structures. Genomic comparison between the species revealed a relatively higher level of divergence in non-coding regions than in coding regions, similar to what has been reported for the genus *Quercus* and *Carya* from the family Fagaceae (Li et al. 2018; Shen et al. 2022). We also identified a considerable number of variations in the noncoding chloroplast genome sequences, such as *rbcL-accD*, *rpl20*-*rps12*, *trnK*-*rps16*, *trnF*-*ndhJ* and *ccsA*-*ndhD*. These noncoding sites may be useful in understanding the ecological significance of the species in terms of spatial distribution and adaptability besides the evolutionary relationship of the new species within Fagaceae.

### ﻿Key to the related species of *Lithocarpusdahuensis*

**Table:** 

1	Female flower solitary	**2**
–	Female flowers in clusters of (2 or)3(–5)	**4**
2	Nut covered with appressed minute hairs	***L.quercifolius***
–	Nut glabrous	**3**
3	Leaf blade margin with 7–10 acute teeth; cupules enclosing 1/4–1/3 of nut	***L.dahuensis***
–	Leaf blade margin with 3–6 obtuse teeth; cupules enclosing the bottom of nut	***L.konishii***
4	Young leaf blade abaxially with tufts of stellate hairs at axils of veins, wall 6–10 mm thick	***L.carolineae***
–	Young leaf blades covered with appressed minute hairs, wall 10–14 mm thick	**5**
5	Leaf blade margin lobate-dentate	***L.cyrtocarpus***
–	Leaf blade margin entire or rarely with 1–3 teeth near apex	***L.gymnocarpus***

## Supplementary Material

XML Treatment for Lithocarpus dahuensis

## Supplementary materials

Supplementary material 1 Supplementary data Data type tables and figures Explanation note The information of the phylogenetic analysis of the species used in the present study (nrITS). Note: “-” indicate the data is not yet public. The information of the phylogenetic analysis of the species used in the present study (plastid genome). Comparison of the LSC, SSC and IR regions among twelve chloroplast genomes of *Lithocarpus* species. Genes are denoted by colored boxes. The gaps between the genes and boundaries are proportional to the distances in bps. Gene nucleotide variability (pi) values of twelve *Lithocarpus* species. The Y-axis shows the pi values; the X-axis shows the genes. https://binary.pensoft.net/file/825994This dataset is made available under the Open Database License (http://opendatacommons.org/licenses/odbl/1.0/). The Open Database License (ODbL) is a license agreement intended to allow users to freely share, modify, and use this Dataset while maintaining this same freedom for others, provided that the original source and author(s) are credited.Miao Zhang, Xiao-Hui Zhang, Shi Shi, Bing-Hua Chen
