# Supplementary material for: ﻿Lithocarpusdahuensis (Fagaceae), a new species from Fujian Province based on morphology and genomic data
Source: PhytoKeys. 2023 Mar 17;222:1–18. doi: 10.3897/phytokeys.222.99370 (PMC10209513; doi:10.3897/phytokeys.222.99370)
Supplement: Supplementary material 1 — Supplementary data [file phytokeys-222-001_article-99370__-s001.doc]

**Appendix A. Supplementary data**

Table S1. The information of the phylogenetic analysis of the species used in the present study (nrITS)

|  | Family | Genus | Species | GenBank accession number |
| --- | --- | --- | --- | --- |
| 1 | Fagaceae | *Lithocarpus* | ***Lithocarpus dahuensis*** | OP957303.1 |
| 2 |  |  | *Lithocarpus konishii* | - |
| 3 |  |  | *Lithocarpus aggregatus* | MF770309.1 |
| 4 |  |  | *Lithocarpus amygdalifolius* | KJ685164.1 |
| 5 |  |  | *Lithocarpus balansae* | EU195795.1 |
| 6 |  |  | *Lithocarpus beccarianus* | AF389101.1 |
| 7 |  |  | *Lithocarpus bennettii* | AF389093.1 |
| 8 |  |  | *Lithocarpus bidoupensis* | KY940070.1 |
| 9 |  |  | *Lithocarpus blumeanus* | AF389091.1 |
| 10 |  |  | *Lithocarpus bullatus* | AY040409.1 |
| 11 |  |  | *Lithocarpus brevicaudatus* | KJ685165.1 |
| 12 |  |  | *Lithocarpus calophyllus* | KJ685166.1 |
| 13 |  |  | *Lithocarpus cleistocarpus* | EF057114.1 |
| 14 |  |  | *Lithocarpus clementianus* | AF389107.1 |
| 15 |  |  | *Lithocarpus coalitus* | MF770305.1 |
| 16 |  |  | *Lithocarpus crassifolius* | MW044410.1 |
| 17 |  |  | *Lithocarpus corneus* | AY040440.1 |
| 18 |  |  | *Lithocarpus cooperatus* | AY040407.1 |
| 19 |  |  | *Lithocarpus conocarpus* | AF389095.1 |
| 20 |  |  | *Lithocarpus chrysocomus* | KJ685167.1 |
| 21 |  |  | *Lithocarpus dahuoaiensis* | KY436002.1 |
| 22 |  |  | *Lithocarpus dealbatus* | LT984601.1 |
| 23 |  |  | *Lithocarpus densiflorus* | AY040370.1 |
| 24 |  |  | *Lithocarpus dodonaeifolius* | KJ685170.1 |
| 25 |  |  | *Lithocarpus echinophorus* | AY040437.1 |
| 26 |  |  | *Lithocarpus echinifer* | AF389089.1 |
| 27 |  |  | *Lithocarpus edulis* | AY040439.1 |
| 28 |  |  | *Lithocarpus encleisocarpus* | AF389094.1 |
| 29 |  |  | *Lithocarpus ewyckii* | AY040413.1 |
| 30 |  |  | *Lithocarpus fenestratus* | MW044411.1 |
| 31 |  |  | *Lithocarpus fenzelianus* | KJ685172.1 |
| 32 |  |  | *Lithocarpus ferrugineus* | AY040414.1 |
| 33 |  |  | *Lithocarpus formosanus* | KJ685173.1 |
| 34 |  |  | *Lithocarpus floccosus* | KP096022.1 |
| 35 |  |  | *Lithocarpus glaber* | AY040435.1 |
| 36 |  |  | *Lithocarpus gigantophyllus* | MF770299.1 |
| 37 |  |  | *Lithocarpus grandifolius* | AY040450.1 |
| 38 |  |  | *Lithocarpus hancei* | MF952868.1 |
| 39 |  |  | *Lithocarpus handelianus* | KJ685185.1 |
| 40 |  |  | *Lithocarpus hatusimae* | AY040410.1 |
| 41 |  |  | *Lithocarpus havilandii* | AF389092.1 |
| 42 |  |  | *Lithocarpus harlandii* | KJ685189.1 |
| 43 |  |  | *Lithocarpus henryi* | EF057110.1 |
| 44 |  |  | *Lithocarpus hongiaoensis* | KY851759.1 |
| 45 |  |  | *Lithocarpus iteaphyllus* | KP092753.1 |
| 46 |  |  | *Lithocarpus keningauensis* | AF389106.1 |
| 47 |  |  | *Lithocarpus kalkmanii* | AF389102.1 |
| 48 |  |  | *Lithocarpus lemeeanus* | MF770306.1 |
| 49 |  |  | *Lithocarpus leptogyne* | AY040416.1 |
| 50 |  |  | *Lithocarpus lepidocarpus* | KJ685194.1 |
| 51 |  |  | *Lithocarpus lampadarius* | AF389099.1 |
| 52 |  |  | *Lithocarpus laoticus* | EU195797.1 |
| 53 |  |  | *Lithocarpus licentii* | MF770301.1 |
| 54 |  |  | *Lithocarpus litseifolius* | KP092755.1 |
| 55 |  |  | *Lithocarpus longipedicellatus* | MF770304.1 |
| 56 |  |  | *Lithocarpus lucidus* | AY040408.1 |
| 57 |  |  | *Lithocarpus luteus* | AF389096.1 |
| 58 |  |  | *Lithocarpus nantoensis* | KJ685197.1 |
| 59 |  |  | *Lithocarpus naiadarum* | KJ685196.1 |
| 60 |  |  | *Lithocarpus nitidinux* | MF952869.1 |
| 61 |  |  | *Lithocarpus nieuwenhuisii* | AY040400.1 |
| 62 |  |  | *Lithocarpus ombrophilus* | MF770297.1 |
| 63 |  |  | *Lithocarpus ochrocarpus* | MF770298.1 |
| 64 |  |  | *Lithocarpus oleifolius* | KJ685198.1 |
| 65 |  |  | *Lithocarpus pachylepis* | AY040442.1 |
| 66 |  |  | *Lithocarpus papillifer* | AY040418.1 |
| 67 |  |  | *Lithocarpus pachyphyllus* | AY040446.1 |
| 68 |  |  | *Lithocarpus palungensis* | AF389103.1 |
| 69 |  |  | *Lithocarpus paniculatus* | KJ685199.1 |
| 70 |  |  | *Lithocarpus pakhaensis* | OL986335.1 |
| 71 |  |  | *Lithocarpus pseudomagneinii* | MF770302.1 |
| 72 |  |  | *Lithocarpus pulcher* | AF389104.1 |
| 73 |  |  | *Lithocarpus rosthornii* | KJ685201.1 |
| 74 |  |  | *Lithocarpus rotundatus* | AF389090.1 |
| 75 |  |  | *Lithocarpus ruminatus* | AF389097.1 |
| 76 |  |  | *Lithocarpus revolutus* | AF389098.1 |
| 77 |  |  | *Lithocarpus rufovillosus* | DQ499087.1 |
| 78 |  |  | *Lithocarpus silvicolarum* | KJ685205.1 |
| 79 |  |  | *Lithocarpus stenopus* | MF770300.1 |
| 80 |  |  | *Lithocarpus skanianus* | MN579529.1 |
| 81 |  |  | *Lithocarpus sericobalanus* | AF389105.1 |
| 82 |  |  | *Lithocarpus shinsuiensis* | KJ685202.1 |
| 83 |  |  | *Lithocarpus taitoensis* | KJ685200.1 |
| 84 |  |  | *Lithocarpus truncatus* | MW044415.1 |
| 85 |  |  | *Lithocarpus turbinatus* | AF389100.1 |
| 86 |  |  | *Lithocarpus uvariifolius* | KJ685212.1 |
| 87 |  |  | *Lithocarpus vinhensis* | MF770303 |
| 88 |  |  | *Lithocarpus vuquangensis* | KY786083 |
| 89 |  |  | *Lithocarpus xylocarpus* | AY040432 |
| 90 | Myricaceae | *Morella* | *Morella rubra* | KP092762.1 |
| 91 | Betulaceae | *Corylus* | *Corylus fargesii* | FJ011741.1 |
| 92 |  | *Carpinus* | *Carpinus cordata* | FJ011715.1 |

Note: “-” indicate the data is not yet public.

Table S2. The information of the phylogenetic analysis of the species used in the present study (plastid genome)

| N0. | Family | Genus | Species | GenBank accession number |
| --- | --- | --- | --- | --- |
| 1 | Fagaceae | *Lithocarpus* | ***Lithocarpus dahuensis*** | OP954095.1 |
| 2 |  |  | *Lithocarpus konishii* | ON422319.1 |
| 3 |  |  | *Lithocarpus litseifolius* | NC_063927.1 |
| 4 |  |  | *Lithocarpus hancei* | MW375417.1 |
| 5 |  |  | *Lithocarpus longinux* | NC_062048.1 |
| 6 |  |  | *Lithocarpus dealbatus* | NC_063459.1 |
| 7 |  |  | *Lithocarpus balansae* | KP299291.1 |
| 8 |  |  | *Lithocarpus fenestratus* | OM112300.1 |
| 9 |  |  | *Lithocarpus polystachyus* | OL569560.1 |
| 10 |  |  | *Lithocarpus cleistocarpus* | OM112296.1 |
| 11 |  |  | *Lithocarpus obscurus* | OM112297.1 |
| 12 |  |  | *Lithocarpus glaber* | MZ750954.1 |
| 13 |  | *Castanea* | *Castanea crenata* | NC_054203.1 |
| 14 |  |  | *Castanea mollissima* | MK352487.1 |
| 15 |  |  | *Castanea henryi* | MH998384.1 |
| 16 |  |  | *Castanea seguinii* | MH998383.1 |
| 17 |  | *Castanopsis* | *Castanopsis sclerophylla* | NC_044680.1 |
| 18 |  |  | *Castanopsis hystrix* | NC_061039.1 |
| 19 |  |  | *Castanopsis carlesii* | NC_057119.1 |
| 20 |  |  | *Castanopsis hainanensis* | NC_037389.1 |
| 21 |  |  | *Castanopsis concinna* | KT793041.1 |
| 22 |  | *Cyclobalanopsis* | *Cyclobalanopsis edithae* | NC_059007.1 |
| 23 |  | *Fagus* | *Fagus crenata* | MH171101.2 |
| 24 |  |  | *Fagus longipetiolata* | MZ562567.1 |
| 25 |  |  | *Fagus engleriana* | NC_036929.1 |
| 26 |  |  | *Fagus hayatae* | MW846258.1 |
| 27 |  | *Quercus* | *Quercus variabilis* | NC_031356.1 |
| 28 |  |  | *Quercus bawanglingensis* | NC_046583.1 |
| 29 |  |  | *Quercus baronii* | MW829651.1 |
| 30 |  | *Trigonobalanus* | *Trigonobalanus doichangensis* | NC_023959.1 |
| 31 | Myricaceae | *Morella* | *Morella rubra* | KY476635.1 |
| 32 | Betulaceae | *Corylus* | *Corylus fargesii* | KX822767.2 |
| 33 |  | *Carpinus* | *Carpinus cordata* | KY312849.1 |


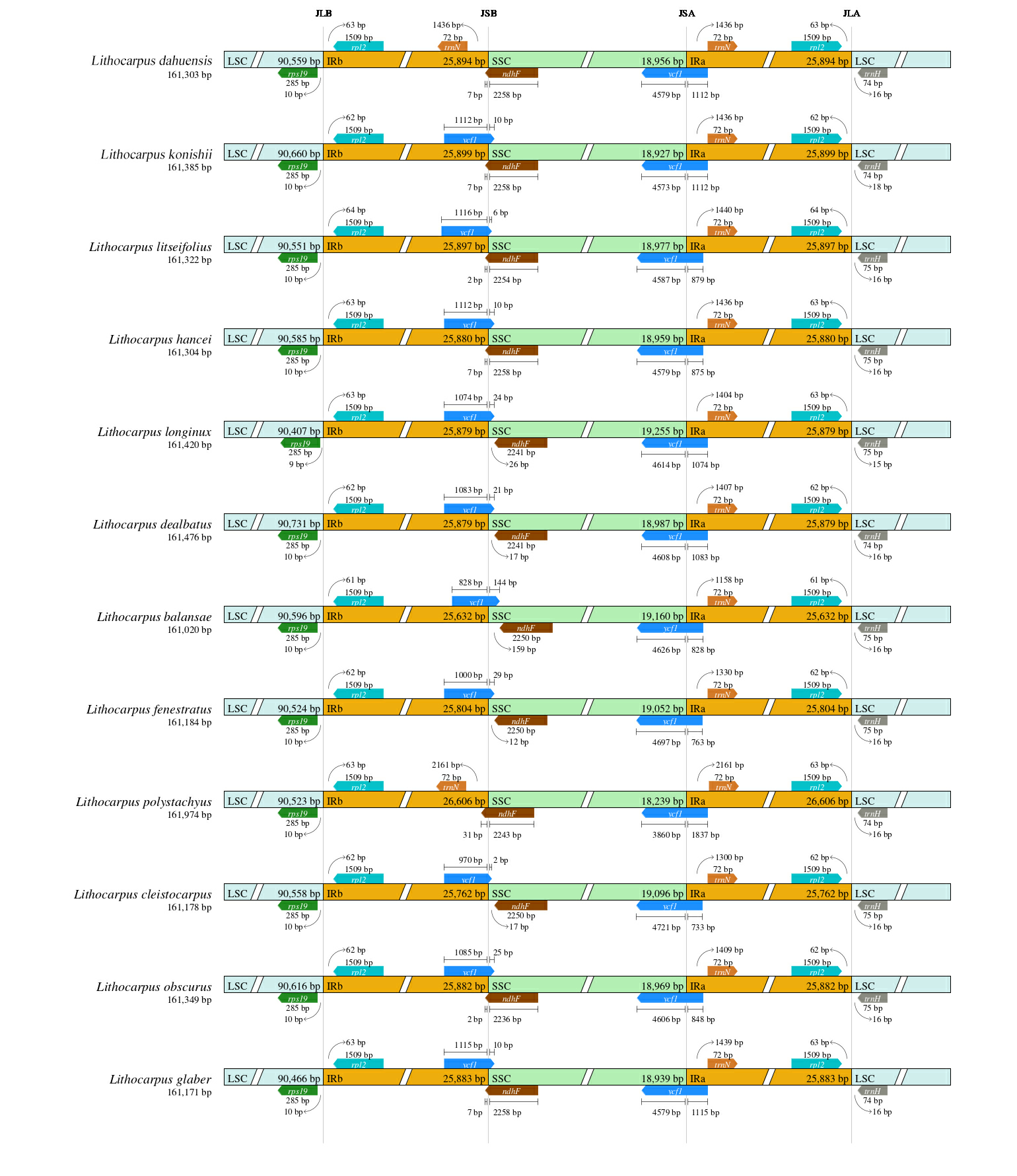


Figure S1. Comparison of the LSC, SSC and IR regions among twelve chloroplast genomes of *Lithocarpus* species. Genes are denoted by colored boxes. The gaps between the genes and boundaries are proportional to the distances in bps.


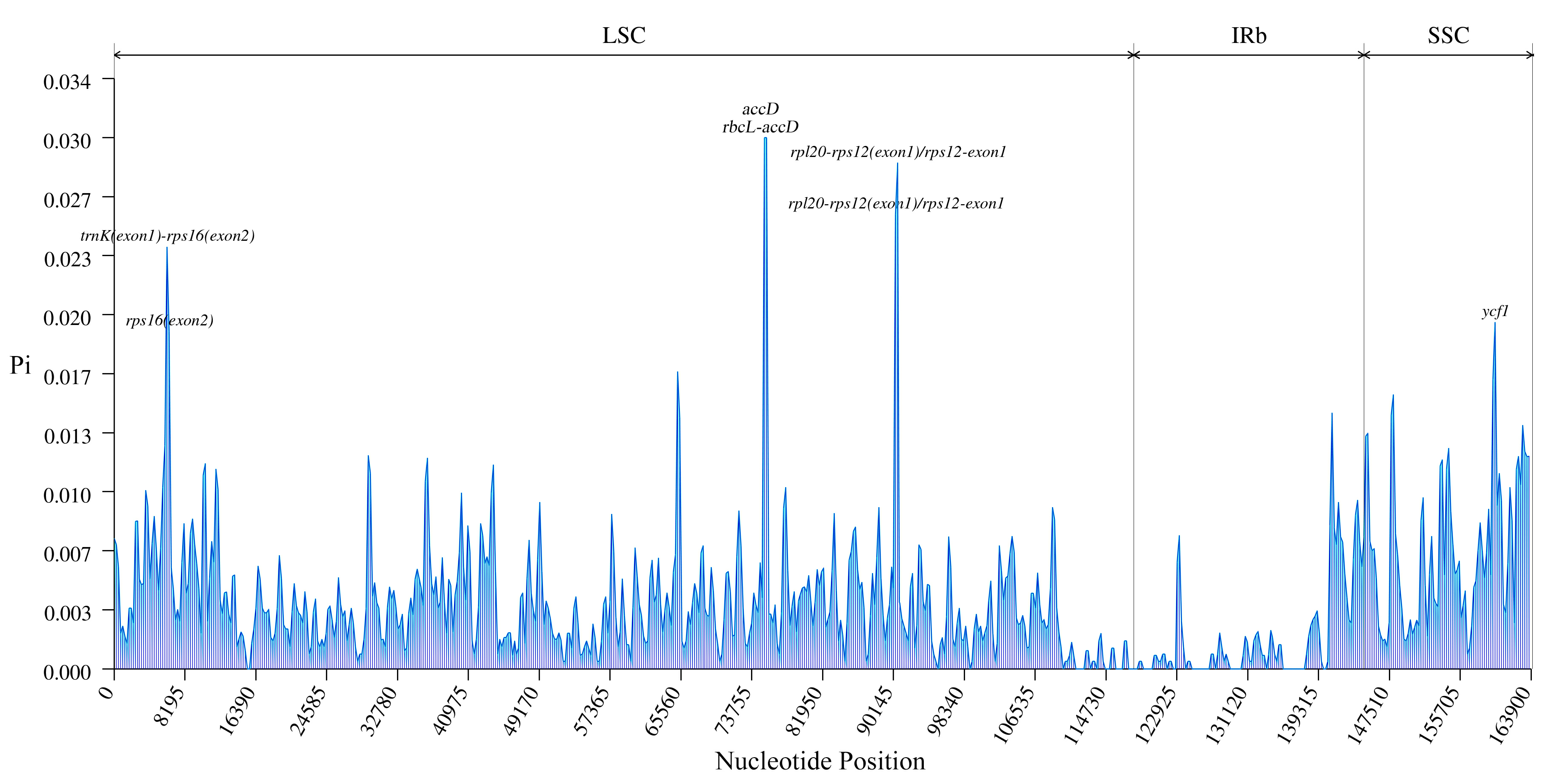


Figure S2. Gene nucleotide variability (pi) values of twelve *Lithocarpus* species. The Y-axis shows the pi values; the X-axis shows the genes.
